# A chromosomal investigation of four species of Chinese Panorpidae (Insecta, Mecoptera)

**DOI:** 10.3897/CompCytogen.v7i3.5500

**Published:** 2013-09-23

**Authors:** Bo Xu, Yankai Li, Baozhen Hua

**Affiliations:** 1 College of Life Sciences, Northwest A&F University, Yangling, Shaanxi 712100, China; 2 Key Laboratory of Plant Protection Resources and Pest Management, Ministry of Education, Entomological Museum, Northwest A&F University, Yangling, Shaanxi 712100, China

**Keywords:** Mecoptera, Panorpidae, chromosome, XO sex-chromosome system, achiasmatic male meiosis

## Abstract

The male adults of four species of the Chinese Panorpidae in Mecoptera were cytogenetically studied using conventional squashing procedures. The results show that their sex-chromosome system belongs to the XO type, with *n* = 19 + X(O) in *Panorpa emarginata* Cheng, 1949 and *Panorpa dubia* Chou & Wang, 1981, *n* = 23 + X(O) in *Panorpa* sp., and *n* = 20 + X(O) in *Neopanorpa lui* Chou & Ran, 1981. X chromosomes of these species usually appear dot-shaped in late prophase I and are easily differentiated from autosomal bivalents. Meiosis in these Panorpidae lacks typical diplotene and diakinesis. In late prophase I, pairs of homologous chromosomes remain parallel in a line and show no evidence of crossing-over. Some of them even appear as a single unit because of extremely intimate association, all with a tendency of increasing condensation. The evolutionary significance of their chromosomal differences and the achiasmatic meiosis of Panorpidae are briefly discussed.

## Introduction

Mecoptera are one of the minor orders of holometabolous insects with approximately 650 described species worldwide (Bicha 2010). They are unique in Holometabola because many taxa of them possess a pair of compound eyes on the head in their larval stages (Byers and Thornhill 1983). In this respect, Mecoptera may represent one of the basal lineages in the Holometabola, or more specifically the most basal taxon of Antliophora (Kristensen 1999).

Panorpidae are the most species-rich family in Mecoptera, with over 420 described species assigned to six genera (Ma et al. 2009, Ma and Hua 2011, Zhong and Hua 2013). They are commonly called scorpionflies because the male genital bulb (the ninth abdominal segment) is enlarged and recurved upward, superficially resembling the stinger of a scorpion (Esben-Petersen 1921). *Panorpa* Linnaeus, 1758 is the largest genus of Panorpidae and is such a diverse taxon that it is often subdivided into different species groups based on external morphological characters for regional faunas (Ma and Hua 2011). The genus *Neopanorpa* Weele, 1909 is an Oriental group in Panorpidae with more than 130 known species in the world to date (Cai and Hua 2009).

The cytogenetics of Mecoptera received a passing interest from the 1930s to the 1970s. To date, only some European and American species have been cytogenetically studied. Species of *Panorpa* were first reported to have an XO sex determination mechanism in males and to have a fairly high complement number (more than 40) by Naville and de Beaumont (1934). Subsequently, male hangingfly *Bittacus italicus* (Müller, 1766) was also reported to have XO sex chromosomes with 13 pairs of autosomes (Matthey 1950). Cooper (1951, 1974) found a different sex determination system in the family Boreidae: *Boreus brumalis* Fitch, 1847 possesses compound sex chromosomes X_1_X_2_Y with 11 pairs of autosomes in males and *Boreus notoperates* Cooper, 1972 possesses XO sex chromosomes with 9 pairs of autosomes in males. Ullerich (1961) found achiasmatic meiosis in three species of *Panorpa*. Later, to elucidate achiasmatic meiosis in *Panorpa*, Gassner (1969) investigated the synaptonemal complex and chromosome structure in the achiasmatic spermatogenesis of *Panorpa communis* Linnaeus, 1758. *Chorista australis* Klug, 1838 (Choristidae) was also found to possess XO sex-chromosome system (Bush 1967). According to cytological observations of spermatogenesis, Atchley and Jackson (1970) found that male scorpionflies of *Panorpa anomala* Carpenter, 1931 and *Panorpa acuta* Carpenter, 1931 have both achiasmatic meiosis and 2*n* = 45 chromosomes, but male hangingflies of *Bittacus pilicornis* Westwood, 1846 and *Bittacus stigmaterus* Say, 1823 (Bittacidae) have chiasmatic meiosis with relatively low chromosome numbers (2*n* = 29 and 31, respectively).

To increase our knowledge of the cytogenetic nature and chromosomal evolution in Mecoptera, we studied meiosis in four species of the Chinese Panorpidae, including three species of *Panorpa* and one more species of *Neopanorpa*.

## Materials and methods

Male adults of *Panorpa emarginata* Cheng, 1949, *Panorpa dubia* Chou & Wang, 1981, *Panorpa* sp., and *Neopanorpa lui* Chou & Ran, 1981 were investigated using conventional squashing procedures. At least three specimens of each species were sampled. The examined species and their localities are listed in Table 1.

**Table 1.** The examined species and their localities.

**Species**	**Localities**	**Collection date**
*Panorpa emarginata*	Taibai Mountain, Shaanxi	Early June 2007
*Panorpa dubia*	Huoditang Forest Farm, Shaanxi	Early June 2012
*Panorpa* sp.	Tongbai Mountains, Henan	Late July 2012
*Neopanorpa lui*	Nangong Mountain, Shaanxi	Middle June 2012

Testes of these species were extracted from ethyl ether anaesthetized specimens and subjected to hypotonic treatment in 0.48% solution of potassium chloride for 15 min, then fixed in a mixture of methanol and acetic acid (3:1) for 2 h. The fixed testes were squashed and stained with 1% Giemsa in Sörense buffer solution (0.067 mol/L, pH 6.8) for 10 min except for *Panorpa emarginata*, which was stained with 2% hematoxylin solution for 10 sec. Photographs were taken with a Nikon DS-Fil digital camera equipped with a Nikon Eclipse 80i microscope.

## Results

### *Panorpa emarginata* Cheng

**Figures 1.**
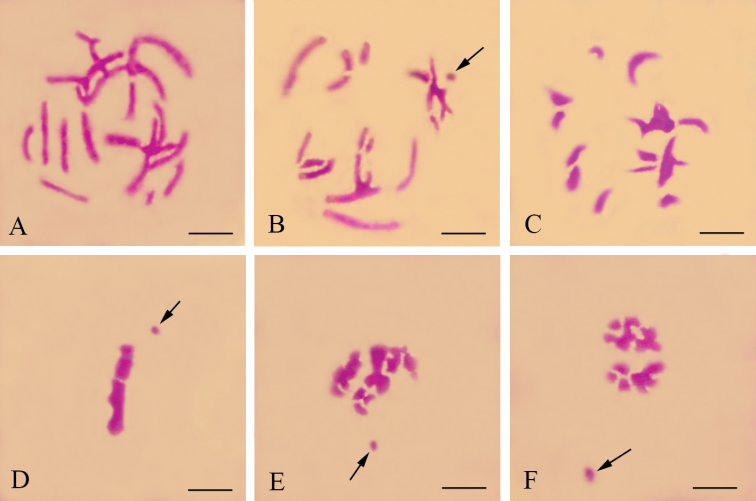
Meiotic chromosomes of male *Panorpa emarginata* subjected to hematoxylin staining. **A** pachytene **B** late prophase I showing the dot-shaped X univalent (arrow) and achiasmatic bivalents **C** metaphase I showing substantially more condensed chromosomes **D** lateral view of metaphase I showing congression of autosomal bivalents and precocity of X univalent (arrow) **E, F** lateral view of anaphase I showing precocity of X univalent (arrow). Bars = 10 μm.

The males have a meioformula of *n* = 19 + X(O) (Fig. 3A). In pachytene (Fig. 1A), the X univalent and autosomal bivalents are all strip-shaped. It is difficult to distinguish the X univalent from autosomal bivalents. In late prophase I (Fig. 1B), the X univalent is dot-shaped and the homologous chromosomes of each bivalent are closely associated. No traces of crossing-over were observed. In pre-metaphase (Fig. 1C), the chromosomes become much more condensed. In the lateral view of metaphase I (Fig. 1D), all bivalents are located in the equatorial plate with the X univalent being precocious. In the lateral view of anaphase I (Figs 1E, 1F), the X can also move ahead of other bivalents.

### *Panorpa dubia* Chou & Wang

**Figures 2.**
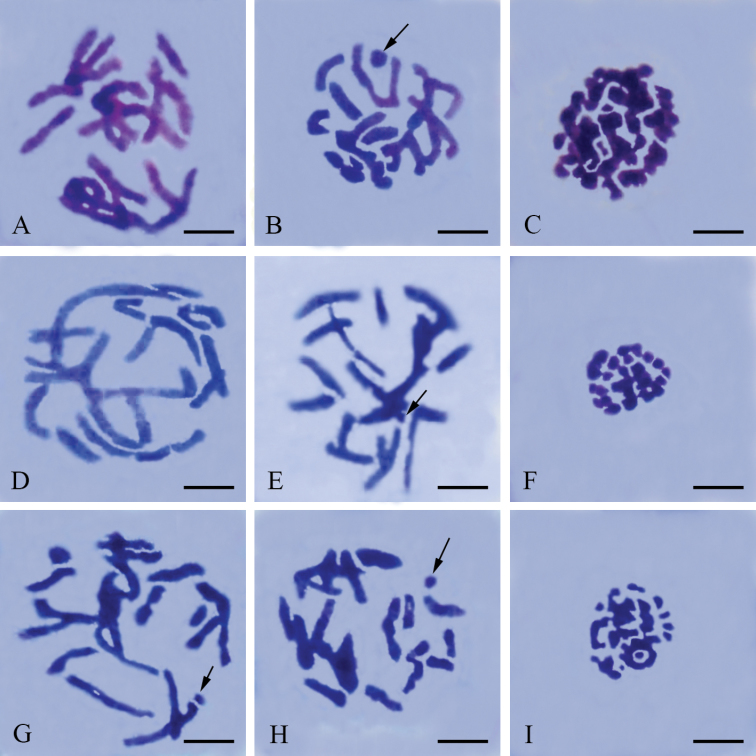
Meiotic chromosomes of male Panorpidae. **A–C** *Panorpa dubia* **D–F** *Panorpa* sp. and **G–I** *Neopanorpa lui* subjected to Giemsa staining **A, D** pachytene **B, E, G, H** late prophase I showing more condensed bivalents than in pachytene and the dot-shaped X univalent (arrow). **C, F, I** polar view of metaphase I. Bars = 5 μm.

**Figures 3.**
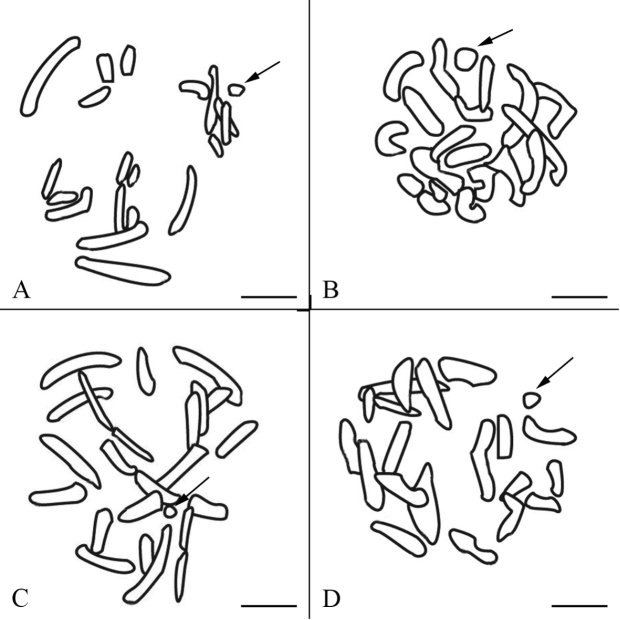
Schematic drawings of late prophase I chromosomes. **A** *Panorpa emarginata* **B** *Panorpa dubia* **C** *Panorpa* sp. and **D** *Neopanorpa lui* corresponding to Fig. **1B**, Fig. **2B**, Fig. **2E** and Fig. **2H**, respectively. The X univalents are indicated by arrows. Bars = 5 μm.

The males also have a meioformula of *n* = 19 + X(O) (Fig. 3B). In pachytene (Fig. 2A) these bivalents are rod-shaped and almost of the same size, but the X univalent is difficult to observe. In late prophase I (Fig. 2B), the bivalents become more condensed and the X univalent appears dot-shaped. As in *Panorpa emarginata*, homologous chromosomes are associated with each other so intimately that they appear as single units. No indication of crossing-over was observed. In the polar view of metaphase I (Fig. 2C), the majority of these bivalents present parallel-arranged homologous chromosomes.

### *Panorpa* sp.

The males have a meioformula of *n* = 23 + X(O) (Fig. 3C). In pachytene (Fig. 2D) the X univalent is difficult to observe. In late prophase I (Fig. 2E), these bivalents become condensed and the X univalent is usually dot-shaped. In particular, some bivalents exhibit two parallel-arranged homologous chromosomes without chiasmata. In the polar view of metaphase I, only a few bivalents present parallel-arranged homologous chromosomes (Fig. 2F).

### *Neopanorpa lui* Chou & Ran

In males, the late prophase I reveals a meioformula of *n* = 20 + X(O) (Figs 2G, 3D). There are 20 bivalents of different sizes. Some of the bivalents present two parallel homologous chromosomes. The X univalent is usually dot-shaped. A more condensed stage without chiasmata was observed near the end of prophase I (Fig. 2H). In the polar view of metaphase I (Fig. 2I), only a few bivalents present parallel-arranged homologous chromosomes.

## Discussion

The present work is the first cytogenetic description of the Chinese mecopteran species. In particular, we obtained first cytogenetic data for a *Neopanorpa* species. All the four examined species of Panorpidae possess a relatively high chromosome number, the males with an XO sex-chromosome system and achiasmatic meiosis.

Based on our study, the meioformula of males is *n* = 19 + X(O) in *Panorpa emarginata* and *Panorpa dubia*, *n* = 23 + X(O) in *Panorpa* sp., and *n* = 20 + X(O) in *Neopanorpa lui*. Their chromosome numbers are very similar to those of the European and American species of Panorpidae, whose meioformula is *n* = 22 + X(O) in *Panorpa communis*, *Panorpa anomala* and *Panorpa acuta*, *n* = 21+ X(O) in *Panorpa cognata* Rambur, 1842, and *n* = 20 + X(O) in *Panorpa germanica* Linnaeus, 1758 (Naville and de Beaumont 1934, Atchley and Jackson 1970). This stability implies a model diploid karyotype of Panorpidae of about 40 chromosomes. The existing small differences in chromosome number between these species likely result from chromosomal rearrangements in the evolutionary history of Panorpidae.

Compared with Panorpidae, other families of Mecoptera possess relatively low chromosome numbers. In Boreidae, males of *Boreus brumalis* have a meioformular of *n* = 11 + X_1_X_2_Y (Cooper 1951) and males of *Boreus notoperates* have a meioformula of *n* = 9 + X(O) (Cooper 1974). In Bittacidae, males of *Bittacus italicus*, *Bittacus pilicornis* and *Bittacus stigmaterus* have meioformulas of *n* = 13 + X(O), *n* = 14 + X(O) and *n* = 15 + X(O), respectively (Matthey 1950, Atchley and Jackson 1970). In Choristidae, males of *Chorista australis* have a meioformula of *n* = 14 + X(O) (Bush 1967). This implies that fissions or duplications could play a significant role in the divergence of Panorpidae.

The precocity of X chromosome in anaphase I and the presence of dot-shaped X chromosome in late prophase I imply an XO sex-chromosome system in the four studied species. The XO sex-chromosome system also occurs in five European and American species of Panorpidae, three species of Bittacidae (Naville and de Beaumont 1934, Matthey 1950, Atchley and Jackson 1970), a species of Choristidae (Bush 1967) as well as in a species of Boreidae (Cooper 1974). *Boreus brumalis* has an extraordinary sex-chromosome system X_1_X_2_Y in males (Cooper 1951). This system could originate via translocation between the X chromosome and one autosome in a species with XO sex-chromosome system. Since multiple sex chromosomes are shared by many species of the order Siphonaptera (Rothschild 1975), which is considered as the sister group of Boreidae (Biliński et al. 1998, Whiting 2002), it is also possible that the ancestors of the Mecoptera had multiple sex-chromosome system as in the Boreidae and Siphonaptera, and the XO sex-chromosome system represents a derived character state.

It is generally acknowledged that the formation of the XO systems is ascribed to Y chromosome degeneration from the XY system in Orthoptera and Heteroptera (White 1951, Grozeva and Nokkala 1996). The process of degeneration of Y chromosome has been well studied in some dipteran and orthopteran species, which have a neo-Y chromosome in the process of differentiation (Castillo et al. 2010, Kaiser and Bachtrog 2010). To date, no XY sex-chromosome system has been found in Mecoptera, and therefore it is premature now to conclude that this process also occurs in this order. If the ancestral sex-chromosome system was of the multiple chromosome type, the formation of XO sex-chromosome system in Mecoptera would not appear so simple. Some complicated rearrangements of chromosomes may have been present during the evolution of sex chromosomes in Mecoptera.

The achiasmatic meiosis in male Panorpidae has been reported several times to date (Ullerich 1961, Atchley and Jackson 1970, Welsch 1973). However, this is not the case in Bittacidae (Ashley and Moses 1980) and Boreidae (Cooper 1974), implying that achiasmatic meiosis is a derived character and can be used for phylogenetic analysis in Mecoptera. Achiasmatic meiosis is not limited to Mecoptera, but has also been discovered in male Orthoptera, Mantodea, Heteroptera, Coleoptera as well as in male Diptera and female Lepidoptera (White 1965, Gassner 1969, Traut 1977, Serrano 1981, Grozeva et al. 2008, McKee et al. 2012), implying its polyphyletic origin in Insecta.

Chiasmata are manifestations of meiotic crossovers, which not only facilitate the exchange of DNA between maternal and paternal chromosomes but also perform the important function of securing physical connections between homologous chromosomes that are essential for their co-orientation and proper disjunction at the first meiotic division (Carpenter 1994, Jones and Franklin 2006). Although some forms of crossover may not result in chiasmata (Moens 1996, Sybenga 1996), two inevitable problems arise in most cases when achiasmatic meiosis occurs: the proper segregation of homologous chromosomes in prophase I and the adaptive significance of these species lacking recombination in one sex.

To solve the first problem, some different modes have been proposed to facilitate the segregation of non-exchange homologous chromosomes. For example, in meiosis of male *Drosophila*, adhesion of homologous chromatids probably contributes to the association of homologous chromosomes (Wolf 1994). The associated chromosomes can be sequestered to discrete pockets of the prophase nucleus to ensure their segregation at meiosis I (Hawley 2002, Vazquez et al. 2002). In Panorpidae, male *Panorpa* has a modified synaptonemal complex with all four chromatid axes being connected by transverse filaments from pachytene to metaphase I, similar to female silk moth *Bombyx mori* Linnaeus, 1758, in which the synaptonemal complex is maintained in an apparently expanded form till metaphase I by a compact layer between homologs, while the oocyte of *Panorpa* contains only two transverse filaments between the axes of the homologous chromatids which disappear before diakinesis (Welsch 1973, Rasmussen 1977).

As far as the second problem is concerned, sexual recombination is generally considered as an adaptive advantage of sexual organisms (Eshel and Feldman 1970, Rice 2002, Carvalho 2003), but many species with halved capacity of recombination exist in the world biota for millions of years. Some mechanisms must therefore counterbalance the seeming disadvantages. White (1973) proposed two alternative explanations: selection for a lower level of recombination or facilitation of paracentric inversion heterozygosity. Altiero and Rebecchi (2003) sustained the first explanation in tardigrades. Serrano (1981) argued that male achiasmatic meiosis in several phylogenetic lineages of Caraboidea (Coleoptera) represented the final step towards coadapted gene blocks that must be preserved from recombination. In Saldidae and Miridae (Heteroptera), achiasmatic meiosis was considered as one of the mechanisms by which regular segregation of homologous chromosomes was achieved, and the reduction of recombination was only a side effect (Nokkala and Nokkala 1983, 1986). In Panorpidae, however, the adaptive significance of the absence of recombination in males remains unclear and needs further investigation.
